# The non-linear electrical properties of human skin make it a generic memristor

**DOI:** 10.1038/s41598-018-34059-6

**Published:** 2018-10-25

**Authors:** Oliver Pabst, Ørjan G. Martinsen, Leon Chua

**Affiliations:** 1 Department of Physics, University of Oslo, Sem Sælands vei 24, 0371 Oslo, Norway; 2 Department of Clinical and Biomedical Engineering Oslo University Hospital, Sognsvannsveien 20, 0372 Oslo, Norway; 3 Department of EECS, University of California, Berkeley, 253 Cory Hall, Berkeley, CA 94720-1770 USA

## Abstract

An electrical measurement is non-linear when the applied stimulus itself affects the electrical properties of the underlying tissue. Corresponding voltage-current plots may exhibit pinched hysteresis loops which is the fingerprint of a memristor (memory resistor). Even though non-linear electrical properties have been demonstrated for different biological tissues like apples, plants and human skin, non-linear measurements as such have not been defined, yet. We are studying the non-linear properties of human skin systematically and initiate non-linear measurements on biological tissues as a field of research in general by introducing applicable recording techniques and parameterization. We found under which voltage stimulus conditions a measurement on human skin is non-linear and show that very low voltage amplitudes are already sufficient. The non-linear properties of human skin originate from the sweat ducts, as well as, from the surrounding tissue, the stratum corneum and we were able to classify the overall skin memristor as a generic memristor. Pinched hysteresis loops vary largely among subjects; an indication for the potential use in biomedical sensor applications.

## Introduction

When a constant low-frequency sinusoidal voltage of high amplitude (e.g., 13 V) is applied to human skin^1^, the shape of the measured current differs from sinusoidal. This observation implies that the measurement is non-linear, and electro-osmosis, the directed motion of liquid caused by an electric field, within the sweat ducts has been suggested as the underlying mechanism^1^. Human sweat contains ions, is therefore highly conductive, and the level of sweating determines the conductance of the skin in the low-frequency range. The level of sweating may be affected by the applied voltage itself, which has an impact on the resulting current (non-linear measurement). Similar observations were done with a current stimulus^2^. The resulting voltage-current (V-I) plots (Lissajous figures) show hysteresis loops with a pinched point in the coordinate origin, which is the “fingerprint” of a memristor (memory resistor)^3^, the fourth passive electrical circuit element^4^. A memristor relates voltage and current via the state-dependent Ohm’s law and a first realization (based on titanium dioxide) was presented in 2008^5^. Current research on memristors includes their realizations based on different materials, such as tantalum oxide^6,7^, zinc oxide^8^ and their applications, for example, in neuromorphic computing^9–11^ or in circuits emulating arithmetic operations^12,13^. However, biological memristors, such as the Venus flytrap^14^ and slime mold memristors^15^, have also been demonstrated. Different memristor types are defined^16^ and a generic memristor, for example, can be described by

1 $$v=M(x)i$$

2 $$\frac{dx}{dt}=f({\boldsymbol{x}},\,i)$$

using memristance *M*(***x***) (in analogy to resistance) where ***x*** is a vector of state variables. A measurement on any memristor is non-linear as soon as its inner state is affected by the applied stimulus.

**Figure 1:**
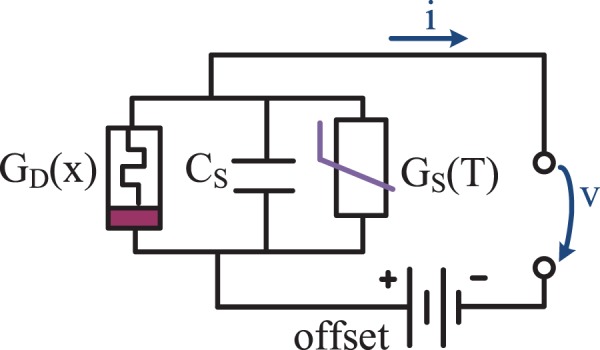
Simple electrical circuit model of human skin in non-linear measurements based on our findings. The sweat duct memristor, with memductance *G*_*D*_(*x*), consists of several sweat ducts, and its ability to conduct current is dependent on sweat duct filling. The state-dependent conductance of the stratum corneum *G*_*S*_(*T*) increases with increasing temperature *T*. The capacitive properties (*C*_*S*_) of the stratum corneum and a DC offset will affect the measurements.

The human skin memristor as previously described^17^ must be labelled more precisely as a “sweat duct memristor”, since there is a second, non-linear mechanism that originates from the stratum corneum^18^, which may be modelled as a memristor itself. The overall skin memristor consists of both memristor types (electrically in parallel to each other) and the expression in terms of memductance (Fig. 1) (non-linear, state-dependent analog of conductance *G*) is useful. It seems that the sweat duct memristor *G*_*D*_(*x*) more or less dominates, as long as there is galvanic contact through the sweat ducts.

The small-signal behavior around an equilibrium state (*x*_*Q*_, *T*_*Q*_) of the parallel circuit (Fig. 1) can be described by the state-dependent mem-admittance (analog to admittance), defined as follows:

3 $$Y({x}_{Q},\,{T}_{Q})=G({x}_{Q},\,{T}_{Q})+jB$$

4 $$=\,{G}_{D}({x}_{Q})+{G}_{S}({T}_{Q})+j\omega {C}_{S},$$

with a susceptance of *B*, an imaginary unit of *j* and *ω* = 2π*f*. The capacitive properties of the stratum corneum (*C*_*S*_) that are related to its humidity^19^ will also affect the measurements, especially at higher frequencies. If the frequency *f* is zero, the memristance at a certain state will be one divided by the corresponding memductance. Natural differences in electrical potentials among skin sites (endogenous skin potentials), as well as, half-cell potentials under the recording electrodes will contribute a direct current (DC) offset to the measurement^20^.

Non-linear properties of human skin and the corresponding recording methods can be allocated within the field of Bioimpedance that encompasses the passive electrical properties of organic tissues^21^. However, the term “Bioimpedance” was only associated with linear measurements, yet, and we suggest the terms “non-linear Bioimpedance”, “state-dependent Bioimpedance” or “Biomempedance” to explicitly state the non-linear part of Bioimpedance.

**Figure 2:**
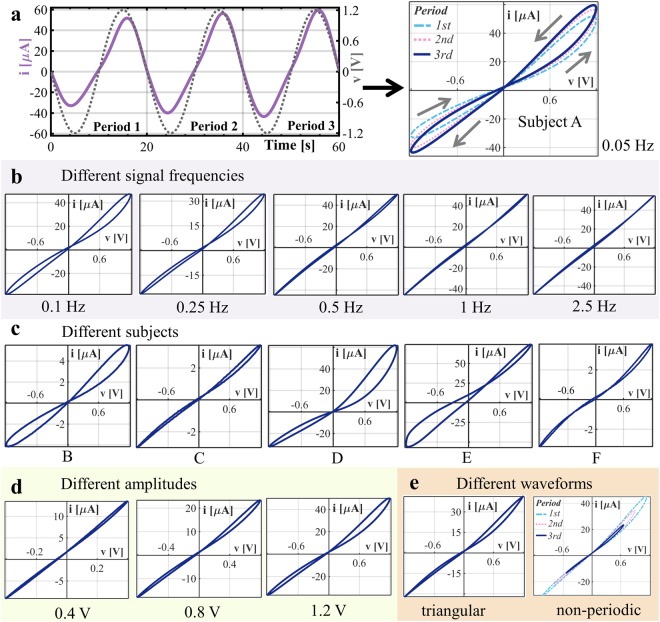
Voltage-current (V-I) plots recorded from the forehead, always for the third period of each applied signal. Each voltage stimulus was continuously applied for three periods, as shown in (**a**) Applied sinusoidal voltage (amplitude of 1.2 V and *f* = 0.05 Hz), measured current *i* over time (left) and the corresponding V-I plot (right) for subject A. (**b**) Same subject and sinusoidal voltage amplitude as in (**a**), but different frequencies. The lobe area of the pinched hysteresis loop decreases with an increasing frequency (applies for all subjects). The presented V-I plot at 1 Hz still shows a pinched hysteresis loop with very small lobes and a large shift in the position of the pinched point. (**c**) Same voltage stimulus as in (**a**), but different subjects. Loops that were symmetric and exhibited a relatively large lobe were observed for 6 subjects at different current levels (compare subject A with subject B) when this voltage stimulus was applied. Loops that instead showed a relatively small area in each lobe (see subject C) were observed for 10 subjects. Asymmetric loops with relatively large lobes in the first quadrant, but small lobes in the third (see subject D) were observed for 8 subjects. The asymmetric shape with a very large shift in the position of the pinched point (see subject E) was unique for. Twenty-five subjects in total (out of 28) showed hysteresis loops with one pinched point (but different shapes, see subjects A to E), which reflects the sweat duct memristor. The remaining three subjects showed highly symmetric hysteresis loops with small lobes and 2 pinched points (see subject F), which is an indication that the stratum corneum NTC thermistor was dominating the measurement. (**d**) The sinusoidal voltage stimulus with *f* = 0.05 Hz but different amplitudes are shown for subject G. The relative lobe area increases with increasing amplitude (applies for all subjects). Pinched hysteresis loops at an amplitude of 0.4 V were only observed for 21 subjects (most of whom presented very small lobe areas). The remaining seven subjects showed a linear relationship. (**e**) Applied voltage waveforms other than sinusoidal are shown for *f* = 0.05 Hz and subject G. When a pinched hysteresis loop was obtained from the recording with a sinusoidal voltage, it was usually also obtained from the recordings with a triangular and non-periodic (sinusoidal signal with decreasing amplitude) waveform for the same subject and signal frequency. The recording with the non-periodic waveform is shown over three periods, and such recordings were only obtained from fifteen test subjects due to instrumentation error.

**Figure 3:**
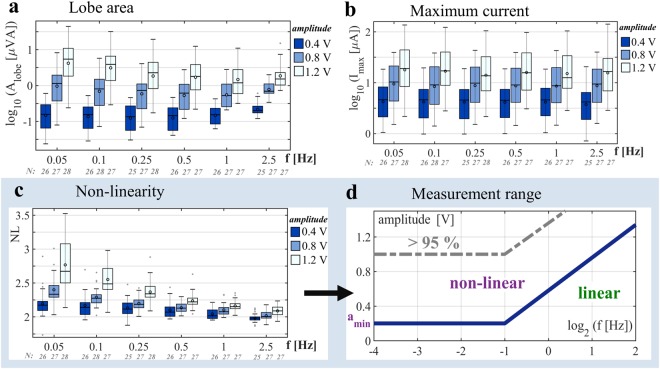
Boxplots for all test subjects recorded from the forehead, for 3^rd^ period of all applied sinusoidal voltages (3 amplitudes, 6 frequencies) provide information about how the V-I characteristics change with amplitude and frequency. The horizontal line in the middle of each boxplot denotes the median; the circle indicates the mean value; and the whiskers indicate the 5% and 95% percentiles. The number *N* of subjects included in the evaluations is provided under each boxplot (see “Statistical analysis” in the methods part). (**a**) Lobe area (logarithm to base 10). The mean and median of the lobe area continuously decrease up to a certain frequency (e.g., up to 1 Hz for 1.2 V amplitude) and then increase above that frequency, when the capacitive properties of the stratum corneum begin to interfere noticeably. (**b**) Maximum current (logarithm to base 10) (**c**) A non-linearity (*NL*) value of 2 implies a linear measurement, and the higher the value of *NL*, the higher the non-linearity of the measurement. The pinched hysteresis loop of the third period in Fig. 2a results in a *NL* value of approximately 3.1, the loop of subject F in Fig. 2c (example with two pinched points) results in an *NL* value of 2.52. Results from the linear mixed effects model analysis (see methods; the number of observations at the forehead was 481) show that the frequency (as logarithm to base 2 in this model) and the absolute value of the amplitude (p-value < 0.001 for both) have significant effects on the non-linearity parameter. The value of the *NL* parameter increases by 0.328 ± 0.039 (95%-CI) with an increase in amplitude of 1 V, and each bisection of the frequency increases the value by 0.075 ± 0.007 in the obtained model. (**d**) Boundary between the non-linear and the linear measurement range (blue line) in the amplitude - frequency plane based on the results in (**c**). This boundary is an approximation and valid for measurements at the forehead with sinusoidal voltage and the chosen methods (e.g. use of dry electrodes). A measurement was considered to be non-linear as soon as the median NL value was larger than 2.05. The area above the grey dotted line is an approximation for a range in which more than 95% of the subjects showed non-linear behavior (based on the lower whiskers of the NL values that were above 2.05).

The non-linear properties of human skin were studied systematically (recordings on 28 test subjects) by the use of a suitable recording system that was previously presented by our group^22^. Recordings from the forehead (Figs 2 and 3), the earlobe (see Supplementary Figs S1 and S2) and the fingertip (see Supplementary Figs S3 and S4) of the same subjects were done simultaneously. Sinusoidal (with amplitudes of 0.4 V, 0.8 V, and 1.2 V), triangular and non-periodic voltage stimuli were applied with six different frequencies (0.05 Hz to 2.5 Hz) each to verify whether human skin is a memristor and under what voltage stimuli conditions a measurement becomes non-linear.

## Results and Discussion

Pinched hysteresis loops (Fig. 2) in the V-I plot were observed for different subjects and voltage stimuli and the results confirm that human skin is a memristor. This property is not a unique phenomenon of single subjects; it is a general property of human skin across different ages, genders, and skin sites (forehead in Fig. 2, earlobe in Supplementary Fig. S1 and fingertip in Supplementary Fig. S3).

### The sweat duct memristor

In the positive half of the period, the sweat is moved by electro-osmosis toward the skin surface, resulting in further filling of the sweat ducts and better conduction pathways (Fig. 2a). Hence, memductance increases. During the negative half of the period, the opposite occurs. Consequently, the orientation of all measured pinched hysteresis loops is counter-clockwise in the first quadrant and clockwise in the third quadrant and the two branches of the loop crossed the pinched point with different slopes (“transversal” pinched hysteresis loop). As the frequency increases, less time remains for the ions to move and the resulting decrease in lobe area (Figs 2b and 3a) is another fingerprint of memristors^23^.

The position of the pinched point is usually very close to the origin of coordinates for applied voltages with frequency of 0.05 Hz and then shifts away with an increasing frequency (Fig. 2b) which can be explained by the capacitive properties of the stratum corneum^20^. Recordings from organic memristors in general contain parasitic elements that cause a shift in the position of the pinched point^3,23^. Any DC offset will also cause a shift in pinched point position^20^. The appearance of the hysteresis loops may change slightly from period to period, as can be seen in Fig. 2a. Possible reasons include the DC offset, and different durations between filling and emptying of the sweat ducts. The changes from period to period are even smaller in the recordings of other subjects or at higher frequencies. Furthermore, the appearance of the hysteresis loops stabilizes with an increasing period number, and the use of the third period for further presentation and analysis was found to be a good trade-off between the final appearance and recording time. However, one has to be aware that since a non-linear measurement will affect the internal state of the underlying tissue, it will have an effect on any measurement that is conducted afterwards (in a series of recordings).

### The stratum corneum memristor

Panescu *et al*.^18^ reported non-linear properties of the stratum corneum that change with temperature which is in accordance with several observations made in this study. Hysteresis loops with two pinched points were recorded from three subjects at the forehead (subject F in Fig. 2c) and from 18 subjects at the earlobe (subjects C and F in Supplementary Fig. S1). The current responses in these recordings were quite small (peaks between 2 µA to 10 µA at 1.2 V amplitude) which is indication that there was little or no galvanic contact through the sweat ducts and, hence, that the recorded current travelled mainly through the stratum corneum (which also contains ionic pathways^24^). The stratum corneum consists of keratinized tissue^21^ and it is shown that the conductance of human hair, which also consists of keratinized tissue, increases with a temperature increase^25^. It is therefore likely that the stratum corneum behaves like a negative temperature coefficient (NTC) thermistor, which can be modelled as a memristor. Small currents (smaller than 10 µA) through the stratum corneum under the measuring electrode might increase the temperature only slightly, but sufficiently high to cause a memductance change. As soon as the current flow stops, the temperature will decrease again. The memductance of the stratum corneum will consequently achieve two maximum states within one period (T) of an applied sinusoidal voltage (at ¼ T and 3/4 T), and the two branches of the loop are touching the pinched point with equal slopes (“tangential”). It is possible to obtain a hysteresis loop with two pinched points if a memristor that exhibits a tangential pinched hysteresis is connected in parallel with a capacitance (done by simulations, unpublished results).

### Range of linear and non-linear measurements and parameterization

Whether a measurement is linear or non-linear is determined by the applied voltage stimulus (Fig. 3c,d) and can be expressed by the non-linearity (NL) parameter (adapted from^26^, see methods). The value of the NL parameter gives indication about the shape of the V-I plot in the first quadrant. If it is a straight line (pure resistive, linear measurement), the NL value is 2. If a state change is happening, both, the sweat duct and the stratum corneum memristor exhibit an increase in the state-dependent conductance within the positive halve period of the applied voltage. Corresponding V-I plots are then non-linear and different from a straight line; and the resulting NL value becomes larger than 2. The measurements tend to become linear with an increase in frequency (Figs 2b and 3c) and decrease in amplitude. The recordings became non-linear already for voltage amplitudes of 0.4 V (valid for all three recorded skin sites: forehead, earlobe, fingertip) which is quite surprising but it is likely that recordings with amplitudes somewhat below 0.4 V (a_min_, Fig. 3d) are just linear (i.e. no significant electro-osmosis occurs). However, signal frequency and amplitude should be chosen carefully for both, linear and non-linear measurements. Electrodermal activity (EDA) recordings^27^ on human skin, as an example, are supposed to be linear but the application of a DC voltage level of 0.5 V (standard method^28^) may already cause a non-linear measurement. To ensure a linear measurement, the alternating current (AC) method of recording EDA should be chosen instead^29^. However, the NL parameter does not reflect the extension of the pinched hysteresis loop and a single-valued function that does not result in a straight line will also have a NL value larger than 2. The lobe area (area within the pinched hysteresis loop) as an additional parameter is therefore useful. The absolute value was used to study how the lobe area as such is affected by the frequency of the applied voltage. It decreases with an increase in frequency as it was shown over the whole population (see Fig. 3a). However, this parameter as it is defined is very much dependent on the amount of current passing through the skin memristor. For example the area of the pinched hysteresis loop of subject A in Fig. 2a is much larger than that of subject B in Fig. 2c. For this reason, the area normalized with the maximum current (and the voltage amplitude in addition if needed) could be used as a parameter for between group comparisons instead of the absolute value of the area. The maximum current (Fig. 3b) is the highest value that the current was achieving which always happened within the first quadrant of the V-I plot. The maximum measured currents seem to be more or less constant among the frequencies (Fig. 3b) and were below 100 µA for all subjects (except one at 1.2 V amplitude), which is much lower than the threshold of perception. This is an important issue for test subject safety in potential clinical and commercial use of the non-linear measurements.

### Human skin, a generic memristor

The measured pinched hysteresis loops were generally not odd-symmetric (Fig. 2), implying that the skin memristor is not an ideal memristor. The V-I relationship at 1 Hz and 2.5 Hz in most of the subjects is a straight line up to a certain magnitude, followed by a small curve. Even though the curve at 2.5 Hz in Fig. 2b is very close to a straight line, it bends slightly above 0.6 V. The interpretation is that the overall skin memristor at the forehead is a generic memristor up to a certain voltage magnitude and then tends to be an extended memristor above this value. The *NL* values in Fig. 3c may confirm this finding. The mean and median *NL* values at 2.5 Hz for amplitudes of 0.4 V and 0.8 V are very close to 2, indicating a straight line (These *NL* values must be evaluated carefully, since a phase shift from the capacitance of the stratum corneum will erroneously decrease the *NL* value, see methods). The difference in shape from a straight line at an amplitude of 1.2 V and frequency of 2.5 Hz (*NL* values are larger than 2) implies that the skin memristor has entered the domain of an extended memristor^16^ defined by *i* = *G*(*x*, *v*)*v*. On the other hand, some subjects still showed pinched hysteresis loops under these stimulus conditions, which will also result in NL values larger than 2. A straight line within the full magnitude of the applied voltage at high frequencies, as observed for some other subjects, indicates generic memristors, described by *i* = *G*(*x*)*v*, where the memductance *G*(*x*) is not a function of the voltage *v*.

### Differences between skin sites

The non-linear properties at the earlobe were comparable with the forehead (see Supplementary Figs S1 and S2), but galvanic contact through the sweat ducts was achieved for fewer subjects (9 of 28) using our methods. The recordings of the remaining subjects consequently reflected the non-linear properties of the stratum corneum (hysteresis loop with two pinched points and small currents). If the electrode was pressed against the earlobe (only for testing), a sudden increase in conductance and a hysteresis loop with one pinched point were observed. However, the initial sweat level is crucial for the appearance of the pinched hysteresis loop, and it was found that the voltage-current relationship at the forearm of one subject changed completely after physical exercise^22^. It is likely that galvanic contact becomes better in a warm and humid environment. The average relative humidity and room temperature in this study were 30.6% (SD of 5.1%) and 21.6 °C (SD of 0.8 °C), respectively.

The skin of the fingertip (see Supplementary Figs S3 and S4) behaved differently from that of the forehead and the earlobe, and the obtained voltage-current relationships were usually less non-linear. Pinched hysteresis loops (if present) exhibited small lobe areas and only one pinched point (in or close to the origin), which confirms that the sweat duct memristor exists at the fingertip as well.

The fingertip (as part of the palmar skin) is an emotionally active site (exhibiting sweating caused by stimulation through the sympathetic and parasympathetic nervous system) that is less sensitive to thermo-regulation^27^. No hysteresis loops with two pinched points were recorded, indicating that any contribution of a stratum corneum NTC thermistor (if present) was negligible. The reasons for this finding include good galvanic contact through the sweat ducts, which was usually achieved at the fingertip, and the approximately 1 mm^27^ thickness of the epidermis (The thickness of the epidermis at other skin sites than palmar and plantar skin sites is much smaller, about 50 to 200 µm).

### Application of the non-linear electrical measurements

The study here provides a reference data set which is helpful for the design of experiments. From the data it is known now when a measurement on human skin is expected to be non-linear. Non-linear electrical properties were observed from all test subjects in this study but the appearance of the pinched hysteresis loops differed greatly among subjects (Fig. 2c). Physiological properties that may affect the shape of the measured current and the hysteresis loop include the number and diameter of sweat ducts, initial sweat duct filling, skin thickness, ion concentrations, the composition of sweat, the pH of the skin, and the moisture content and the thickness of the stratum corneum. More experiments are needed to verify these assumptions and see how each of these physiological proprieties affects the pinched hysteresis loop; measurements on a human skin model could be useful for this. As stated before, a non-linear electrical measurement can affect the initial state and consequently the results of the subsequent recording. This has to be taken into count in experimental design and biomedical sensor applications. As part of a standardized procedure, it could be useful to do an initial non-linear measurement with, for example, an applied voltage of 1.2 V amplitude and 0.05 Hz over three periods. Furthermore, it is possible to measure small signal (linear) susceptance and conductance before and after the non-linear measurements (as it was done here). This will give additional information that help understanding the non-linear properties. Finally, studies that compare the non-linear electrical properties of a distinguished group (for example test subjects that exhibit a specific disease) with a control group will give additional insights with potential use in diagnostics.

## Methods

### Experimental procedure

#### Subject recruitment, approval

A total of 28 test subjects (16 male, 12 female, mean age 31 years, SD = 9.5 years) were recruited and gave informed consent for participation in the study. A 29^th^ test subject was recruited but the subject’s skin was initially covered by body lotion, and the collected data were excluded from further analysis. The study was performed in accordance with the guidelines given by the South-Eastern Review Board (REC South East) of the Regional Committees for Medical and Health Research Ethics in Norway. Not falling under the definition of clinical research, these guidelines do not pose any requirement for applying for approval of the study protocol, but the research group is responsible for making sure that adequate measures have been taken to ensure electrical safety for the test subjects. This was done a.o. by using a Noratel - IMEDe 1000 ® Medical Transformer system. The measurements were conducted at the University of Oslo in November and December in 2016.

#### Experimental Design

It was randomly chosen whether the measurements were performed on the side of the preferred or non-preferred hand. Electrodes were put in place before the experiment started, and a test measurement was performed. If only noise was measured in one channel, the corresponding electrode was re-attached. Room humidity and temperature were measured before the experiment started.

**Figure 4:**
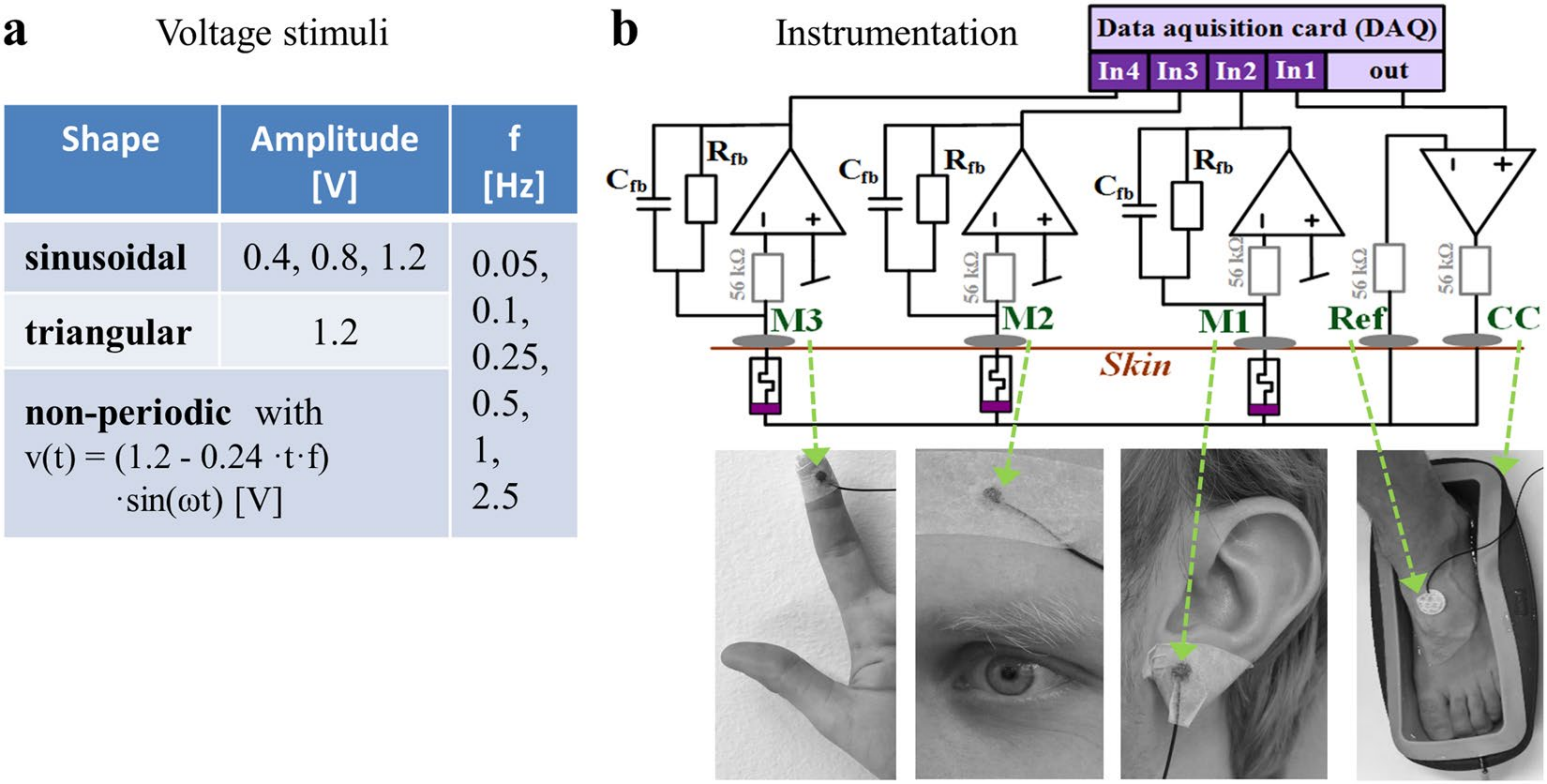
Experimental procedure. (**a**) Voltage stimuli used in the experiment. Five voltage stimulus types (sinusoidal with amplitudes of 0.4 V, 0.8 V, and 1.2 V, triangular and non-periodic) were applied with six different frequencies (0.05 Hz to 2.5 Hz), each. (**b**) Schematic representation of the selected measurement system and the corresponding electrode placement (shown for the left-hand side). The electrode setup on the right-hand side was equivalent. The shown instrumentation is based on a three-electrode configuration^30^, where “CC” is the current-carrying electrode, and “Ref” is the reference electrode. The three measurement electrodes M1, M2 and M3 were connected to the earlobe, the forehead and the fingertip respectively.

In total, 30 different voltage stimuli were used in the experiment (Fig. 4a) and each stimulus was applied for three periods. The sign of each applied voltage stimulus was randomized. Furthermore, the order of the applied voltage stimulus types was randomized. For each voltage stimulus type, a frequency sweep over all six frequencies in a randomized order was applied. The waiting time before a stimulus with a new frequency was applied was 1 second. The waiting time before a run with a new voltage stimulus type was 2 seconds. Small signal admittance (conductance and susceptance) was measured at the same skin sites before (for 30 seconds) and after (for 60 seconds) the experiment.

#### Instrumentation

A custom-built measurement system (see Fig. 4b and further information in^22^) enabled the recording at three different skin sites (under measuring electrodes M1, M2 and M3) at the same time. A data acquisition card (DAQ) (type USB-6356 from National instruments) enabled the application of a constant voltage and simultaneous reading. The DAQ was connected to a personal computer; all powered by an international medical isolation device (IMEDe 1000 from Noratel AG, Germany), to ensure physical separation between test subjects and the mains. The software that controls the DAQ was written in NI LabVIEW (version 2014). Voltage generation and reading were performed with 500 samples per period. The generated voltage was provided at the “out” port, which was directly connected to the input channel “In1” to measure the delay from signal generation inside the DAQ to actual provision by the analog-to-digital converter at the “out” port. In each recording channel, a transimpedance amplifier was used to convert the current through the skin into a voltage that could be read by the DAQ (inputs “In2”, “In3” and “In4”). The feedback resistor *R*_*fb*_ of each transimpedance amplifier has a value of 56 kΩ, and in combination with a small capacitance *C*_*fb*_ (here, 4.7 nF) in parallel, it additionally functions as a low-pass filter to reduce noise.

The three-electrode configuration^30^, enables monopolar recordings under the measurement electrodes. The skin under the current-carrying and reference electrodes does not contribute to the measurement and the voltage is basically applied from deeper layers of the skin (under the measurement electrodes) to the skin surface.

The small signal admittance measurements before and after the experiment were performed with a sinusoidal voltage with an amplitude of 100 mV and a frequency of 20 Hz. The admittance can be separated into the real part (conductance) and the imaginary part (susceptance) via the lock-in technique^21^. The instrumentation is capable of recording approximately two conductance and susceptance values per second.

#### Electrode placement

All electrodes (Fig. 4b) were placed on the same side of the body to avoid current paths through the heart.

The measurement electrodes (prewired, dry Ag/AgCl electrodes from Wuhan Greentek PTY LTD, with an active area of 0.283 cm^2^) were taped to the skin. Dry electrodes were used to exclude any possible influence of ionic gel on the measurements (see^22^). The electrodes were cleaned with ethanol for reuse. One measurement electrode was placed at the earlobe and another at the fingertip of the pointing finger. The third measurement electrode was placed at the forehead above the iris of the eye of the chosen side, at approximately the width of two fingers above the eyebrow.

Compared with the measurement electrode, the choice of the reference electrode is less critical. A prewired Ag/AgCl electrode (type: Kendall 1050NPSM) that was initially covered with solid hydrogel and had an active area of 5.05 cm^2^ was used as a reference electrode to ensure good electrical contact. A saline solution was used as the current-carrying electrode to implement a very large electrode, which will reduce the voltage that must be supplied by the operational amplifier in the three-electrode setup. The foot was chosen to be placed in the saline solution since it is a comfortable setup, and the reference electrode was placed on top of the foot, which was not covered by the saline solution.

### Quantitative analysis

#### Non-linearity

**Figure 5:**
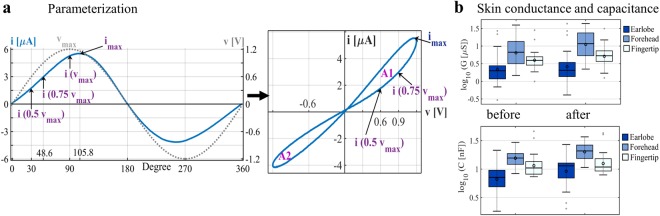
Quantitative analysis. (**a**) One period of the measured current *i* and applied voltage *v* (*f* = 0.05 Hz) for an example measurement and the corresponding V-I plot. Current values that are used for the calculation of non-linearity are illustrated, as is the lobe area, which is the sum of *A1* and *A2*. (**b**) Boxplots of small-signal skin conductance and capacitance for all subjects (logarithm to base 10, each boxplot is based on the evaluation of *N* = 28 subjects), directly before and approximately 1 min after the experiment.

A measure of “non-linearity” (*NL*) was previously introduced^26^ and used to characterize technical memristors; it is defined as the current at *v*_*max*_ to the current at 0.5 *v*_*max*_. A quantitative evaluation of non-linearity in biological memristors is useful as well but an adaption is needed. DC offsets in the measurement (see Fig. 1) will affect the non-linearity value as it has previously been defined^26^. To correct for DC offsets, a slightly different definition of non-linearity is presented in this paper:

5 $$NL=\frac{{i}_{max}-i(0.5\,{v}_{max})}{i(0.75\,{v}_{max})-i(0.5\,{v}_{max})}$$

where *v*_*max*_ is equal to the amplitude of the applied sinusoidal voltage. The current values are illustrated in Fig. 5a, in which one period of an actual measurement is presented. The current *i* has a small DC offset and may exhibit a very small phase shift compared with the applied voltage, but the maximum current value *i*_*max*_ (left side of Fig. 5a) shifts noticeably along the x-axis (at approximately 105.8 degrees). This shift can be explained by the state change of the skin memristor. At 90 degrees, the applied sinusoidal voltage starts to decrease slowly, but the state of the memristor changes further, resulting in a continuing increase of the current. To take this aspect of non-linearity into account, *i*_*max*_ is preferred over *i*(*v*_*max*_) in Eq. (5). If the measured current is linear (and without any phase shift), *NL* becomes 2, in analogy to the parameter in^26^. As the value of *NL* becomes greater, the measured current becomes non-linear. The rise of the loop of the human skin memristor becomes larger with an increasing voltage, as can be observed in Fig. 5a. The increase in current from 0.5 *v*_*max*_ to 0.75 *v*_*max*_ will be smaller than the increase of the current from 0.75 *v*_*max*_ to its maximum *i*_*max*_, and *NL* becomes greater than 2 as a consequence. A phase shift between voltage and current, on the other hand, will misleadingly reduce the *NL* value, since the current is ahead (it would also affect the non-linearity as it is defined in^27^). At very low frequencies, such as 0.05 Hz, the effect is negligible (see estimated phase shifts below), but with an increasing frequency, *NL* values significantly smaller than 2 become possible. Assuming a linear current at 2.5 Hz and two phase shifts of 2.1 and 13.26 degrees as an example, *NL* would be 1.93 in the former case and 1.60 in the latter. Signal noise will also affect the *NL* value.

#### Lobe area

The total lobe area *A*_*lobe*_ (*A1* + *A2*) in the V-I plot (Fig. 5a) is another quantitative measure used in this study. For example, rough approximation for *A1* is to calculate the areas under the upper and lower branches of the loop in the first quadrant and obtain the difference between the two. The difference would have different signs before and after the pinched point and lead to an error. To correct for this error, the absolute values of the differences were calculated in a stepwise manner (from sample to sample) and then summed. The area under the current was calculated via trapezoidal numerical integration using the “trapz” function in MATLAB (version 2016b, academic license). Log transformation (to the base 10) of the total area *A*_*lobe*_ was used to decrease the skewness among test subjects.

#### Statistical analysis

The linear mixed effects model obtained for the non-linearity parameter was applied using the fitlme() function in MATLAB (version 2016b, academic license) with the subject as a random effect (random intercept). The voltage amplitude (absolute value) and frequency (logarithm to base 2) were fixed effects, and non-linearity was the independent variable in the model. The evaluation was performed over the third period of each applied sinusoidal voltage stimulus and separately for the three different skin sites. The number of subjects *N* included in each evaluation (provided under each boxplot) usually differs from 28 since noisy current responses were excluded from the evaluation if *i*_*max*_ was below a certain threshold (2.1 µA, 1.4 µA and 0.7 µA for the measurements with amplitudes of 1.2 V, 0.8 V and 0.4 V, respectively). Furthermore, the response to sinusoidal voltage with an amplitude of 0.4 V was not recorded from 2 subjects at 2.5 Hz and 1 subject at 0.25 Hz due to instrumentation error.

#### Phase shift estimation

A phase shift between the applied voltage and the measured current originates in the capacitive properties of the stratum corneum and will affect the position of the pinched point^20^ and the *NL* value. The phase shift *α* of a simple resistor - capacitor circuit (within the linear range) can be calculated as follows:

6 $$\alpha ={\tan }^{-1}\frac{2{\rm{\pi }}fC}{G}.$$

To obtain an estimation in which range *α* occurs during the experiment, the (linear) susceptance and conductance were measured both before and after the experiment (Fig. 5b). As an example, if *G* is 6.7 µS and *C* is 15.6 nF (median values of the forehead before the experiment), *α* becomes 0.04 degrees for a frequency equal to 0.05 Hz and 2.1 degrees for a frequency equal to 2.5 Hz. If the capacitance is, for example, 30 nF and the conductance is approximately 2 µS (as an extreme example within the range), the phase shift would be 0.27 degrees at *f* = 0.05 Hz and 13.26 degrees at *f* = 2.5 Hz. The actual phase shifts within the non-linear measurements in the experiment are slightly different since the level of the measured conductance at 20 Hz is slightly greater than that measured with a DC voltage^29^ or a very low AC voltage such as 0.05 Hz. Furthermore, since the skin memductance changes during the non-linear measurements, the phase shift also changes as a consequence. A modification of Eq. (6) would be as follows:

7 $$\alpha (x,T)={\tan }^{-1}\frac{2\pi fC}{G(x,\,T)},$$

where *G*(*x*, *T*) is the state-dependent conductance, with *x* and *T* as the inner states that are affected by the applied voltage stimulus.

## Electronic supplementary material

Supplementary Information

## Data Availability

The recorded data from the experiment have been deposited with figshare. These data can be obtained free of charge from https://figshare.com/s/4ad62b29376137ed107c. Custom MATLAB code that was developed for calculation of the parameters (including non-linearity) are available from the corresponding author upon reasonable request.
