# Supplementary material for: The non-linear electrical properties of human skin make it a generic memristor
Source: Sci Rep. 2018 Oct 25;8:15806. doi: 10.1038/s41598-018-34059-6 (PMC6202368; doi:10.1038/s41598-018-34059-6)
Supplement: Supplementary file 1 — Supplementary Information [file 41598_2018_34059_MOESM1_ESM.pdf]

Supplementary Information for

The non-linear electrical properties of human skin make it a generic memristor

Oliver Pabst, Ørjan G. Martinsen and Leon Chua

Corresponding author: Oliver Pabst
Email: oliverpa@mail.uio.no

This PDF file includes: Figs. S1 to S4

A. Data from the earlobe

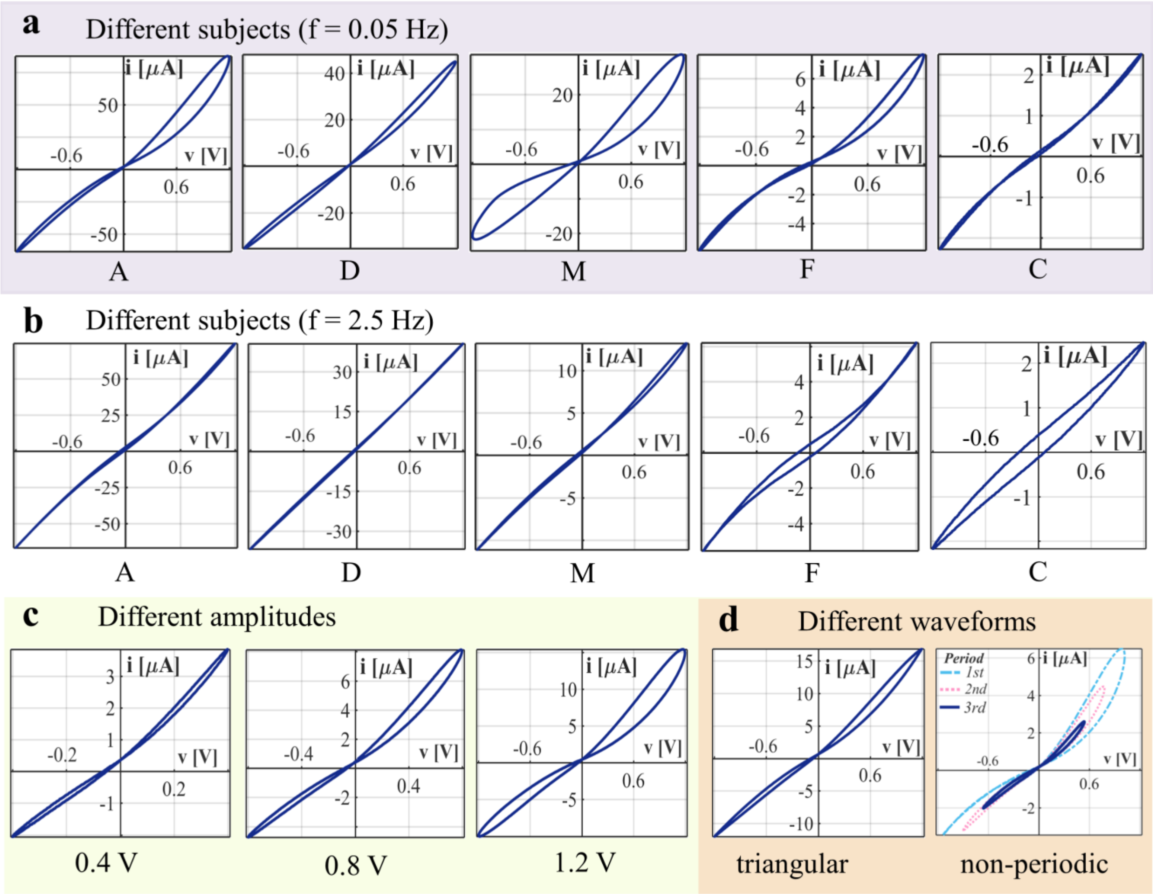

Fig. S1 | Voltage-current (V-I) plots recorded from the earlobe, always for the third period of each applied voltage stimulus. (**a**) Recordings of 5 different subjects (A, D, M, F, and C) with an applied sinusoidal voltage with an amplitude of 1.2 V and frequency of 0.05 Hz. Galvanic contact through the sweat ducts was only achieved for 9 subjects. Five of those subjects showed hysteresis loops with one pinched point and an asymmetric shape, with a very small lobe in the third quadrant (see subject A). Highly symmetric hysteresis loops with a relatively large lobe (total of 2 subjects) (see subject M) and relatively small lobe (total of 2 subjects, see subject D) were observed as well. Galvanic contact through the sweat ducts was not obtained for 18 subjects, since those subjects showed hysteresis loops with two pinched points and a small maximum current similar to those of subjects C and F. The electrode at the earlobe of one subject was not well attached, and electrical contact was not achieved to any extent. (**b**) Same applied signal and subjects as in (a), but a different signal frequency (2.5 Hz). The lobe area of the pinched hysteresis loop decreases with increasing frequency (applies for all subjects). Some subjects showed a pinched hysteresis loop even at 2.5 Hz (see subject M). An example of a hysteresis loop that develops toward a clear straight line is shown for subject D. (**c**) The sinusoidal voltage signal at f=0.05 Hz with different amplitudes is shown for subject N. The relative lobe area increases with an increasing amplitude (applies for all subjects). Hysteresis loops with one pinched point (and with very small lobe areas) were observed for 8 subjects when an amplitude of 0.4 V was applied. Subjects that showed pinched hysteresis loops with two pinched points when the amplitude was 1.2 V exhibited linear voltage current relation when the amplitude was 0.4 V, indicating that an amplitude of 0.4 V is too low to change the state of the stratum corneum thermistor of the earlobe. (**d**) Applied voltage waveforms other than sinusoidal are shown for f=0.05 Hz and subject N. As soon as a pinched hysteresis loop was obtained from the recording with a sinusoidal voltage, a triangular and non-periodic (sinusoidal signal with decreasing amplitude) waveform was obtained. The recording of non-periodic waveform is shown over three periods, and such recordings were only obtained from fifteen test subjects, due to instrumentation error.

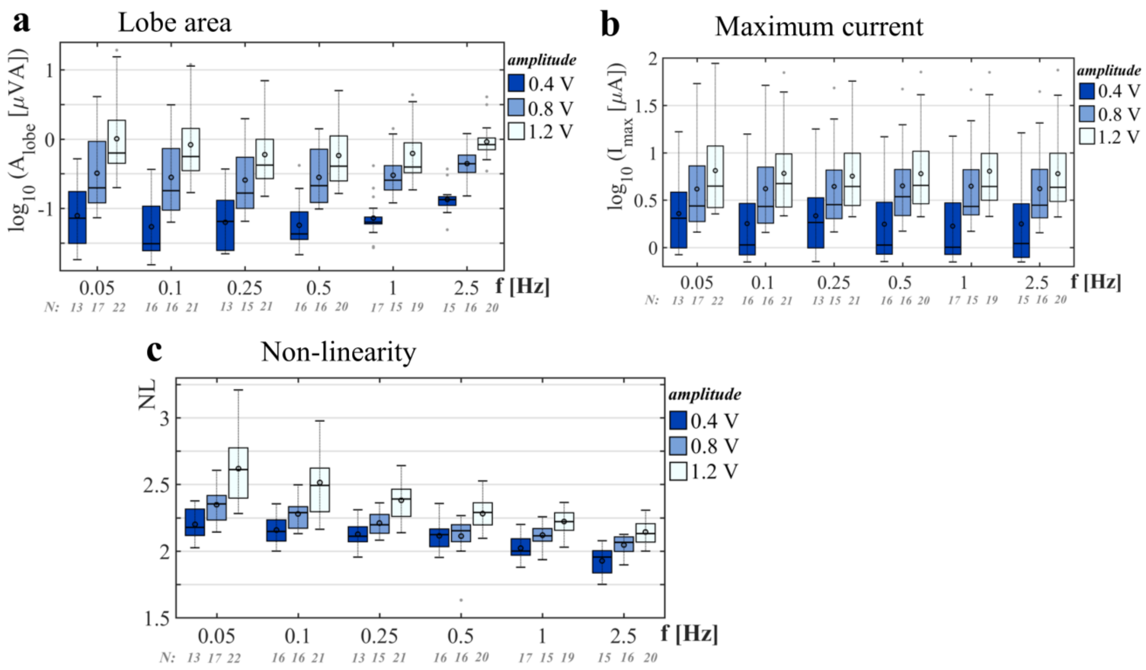

Fig. S2 | Boxplots for all test subjects recorded from the earlobe, for the 3rd period of all applied sinusoidal voltages (3 amplitudes, 6 frequencies). The plots provide information on how the V-I characteristics change with the amplitude and frequency. The horizontal line in the middle of each boxplot denotes the median; the circle indicates the mean value; and the whiskers indicate the 5% and 95% percentiles. The number *N* of subjects, included in the evaluations, is provided under each boxplot (see "Statistical analysis" in the methods part). The recording for one subject at 0.8 V with a frequency of 0.5 Hz (3rd period) was additionally excluded here because the electrode was accidentally touched. (**a**) Lobe area (logarithm to base 10). The mean and median of the lobe area continuously decrease up to a certain frequency (e.g., up to 1 Hz for the median and up to 0.5 Hz for the mean for a 1.2 V amplitude) and increase above that frequency, when the capacitive properties of the stratum corneum start to interfere noticeably. (**b**) Maximum current (logarithm to base 10), (**c**) A non-linearity (*NL*) value equal to 2 implies a linear measurement (straight line in the V-I plot), and the higher the value of *NL*, the higher the non-linearity of the measurement. A single-valued function that does not result in a straight line will also have a value larger than 2. Results from the linear mixed effects model analysis (the number of observations was 307) show that the frequency (as the logarithm to base 2 in the applied model) and the absolute value of the amplitude (p-value < 0.001 for both) have significant effects on the non-linearity parameter. The value of the *NL* parameter increased by 0.360 ± 0.036 (95%-CI) with an increase in amplitude by 1 V, and each bisection of the frequency increased the value by 0.062 ± 0.006 in the obtained model.

B. Data from the fingertip

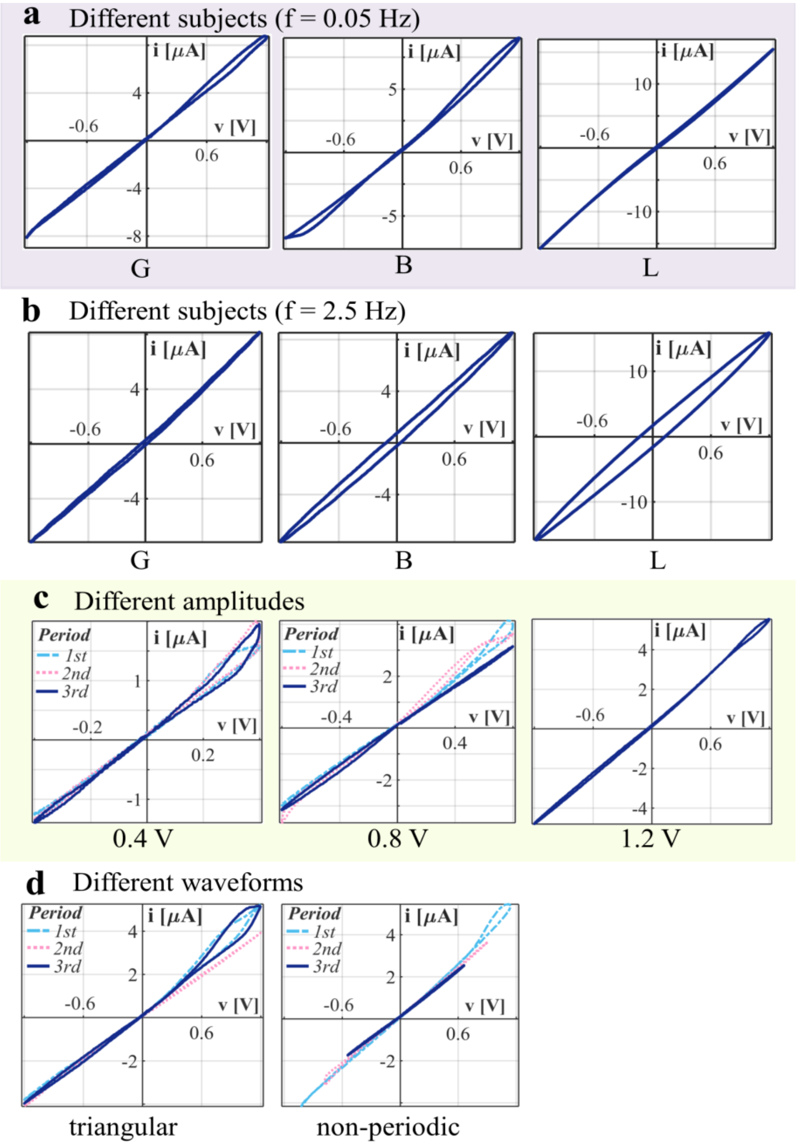

Fig. S3 | Voltage-current (V-I) plots recorded from the fingertip, always for the third period of each applied voltage stimulus. (**a**) Recordings of 3 different subjects (G, B, and L) with an applied sinusoidal voltage with an amplitude of 1.2 V and frequency of 0.05 Hz. The recordings from the fingertip are distinct from those from the earlobe and forehead. The recordings of 15 test subjects show pinched hysteresis loops with very small lobes, as observed for subjects G and B, or even smaller. No pinched hysteresis loop and (almost) linear measurements (see subject L) were obtained for the other 13 subjects. No hysteresis loop with two pinched points was observed. (**b**) Same applied signal and subjects as in (a), but a different signal frequency (2.5 Hz). The capacitive properties dominate in most recordings from the fingertip at 1 Hz, 2.5 Hz or even 0.5 Hz, and an elliptic shape (see subjects B and L) can be observed. (**c**) The sinusoidal voltage signal with *f*=0.05 Hz, but different amplitudes is shown for subject O. The lobe area does not necessarily increase with an increasing amplitude, as demonstrated in this example. The recording at 0.4 V also shows the largest *NL* value and appears to be stable over the three periods shown, which is an indication that electro-osmosis has actually occurred. This result is in accordance with the boxplot shown in Fig. S4c. On the other hand, pinched hysteresis loops for an amplitude of 0.4 V were only observed for 8 subjects, most of whom presented very small lobe areas. The recordings for subject O at 0.8 V differ greatly between the periods, which might be an indication of interference from emotional sweating. The plots of the three periods of recording with an amplitude of 1.2 V (only the third period is shown) are very close together, but only the third period shows a pinched hysteresis loop. (**d**) Applied voltage waveforms other than sinusoidal are shown for *f*=0.05 Hz and subject O. Only the second and third periods of the recording with a triangular waveform show a pinched hysteresis loop. A pinched hysteresis loop was only obtained in the first period of the recording from subject O with a non-periodic waveform. Recording with a non-periodic waveform was only carried out for fifteen test subjects, due to instrumentation error.

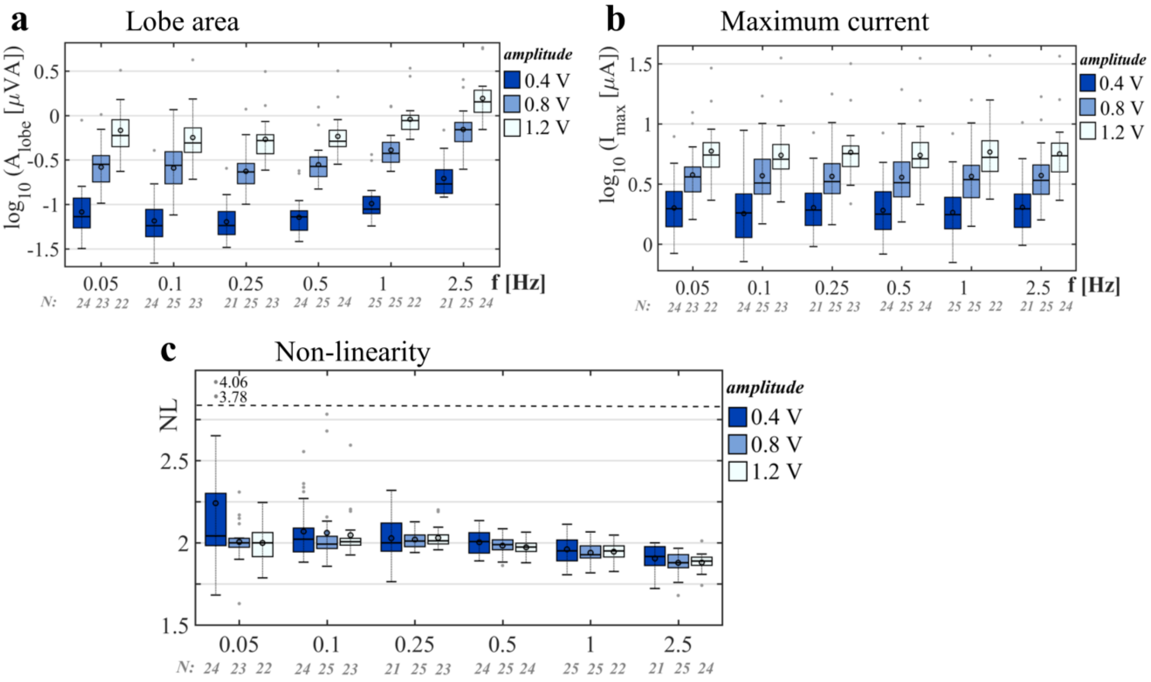

Fig. S4 | Boxplots for all test subjects recorded from the fingertip, for the 3rd period of all applied sinusoidal voltages (3 amplitudes, 6 frequencies). The plots provide information on how the V-I characteristics change with the amplitude and frequency. The horizontal line in the middle of each boxplot denotes the median; the circle indicates the mean value; and the whiskers indicate the 5% and 95% percentiles. The number *N* of subjects included in the evaluations is provided under each boxplot (see "Statistical analysis" in the methods part). (**a**) Lobe area (logarithm to base 10). The increase in area from 0.25 Hz to 2.5 Hz can be explained by the capacitive properties, which start to have a significant effect at those frequencies. (**b**) Maximum current (logarithm to base 10). (**c**) A non-linearity (*NL*) value equal to 2 implies a linear measurement (straight line in the V-I plot), and the higher the value of *NL*, the higher the non-linearity of the measurement. The *NL* values from the fingertip are much smaller than those from the forehead and earlobe, reflecting the observation that many recordings are (almost) linear. The highest *NL* values are obtained with a voltage amplitude of 0.4 V, which is in accordance with the example shown in Fig. S3c. This observation differs from the results for the forehead and earlobe. The extreme values of 3.78 (corresponding to subject O, see Fig. S3c) and 4.06 at 0.05 Hz with an amplitude of 0.4 V reflect actual measured pinched hysteresis loops. Other recordings outside the whiskers may originate from signal noise or emotional sweating. Results from the linear mixed effects model analysis (the number of observations was 425) show that the frequency (as logarithm to base 2 in the used model, p-value < 0.001) and the absolute value of the amplitude (p-value=0.006) have significant effects on the non-linearity parameter. The value of the *NL* parameter decreased by 0.070±0.050 (95%-CI) with an increase in amplitude of 1 V. The decrease in the *NL* value with an increase in amplitude is opposite the results obtained from the earlobe and forehead. Each bisection of the frequency increases the *NL* value by 0.035 ± 0.008 in the obtained model.
